# Racial disparities in use of syringe service programs in King County, WA: a comparison of two cross-sectional surveys

**DOI:** 10.1186/s12954-023-00868-w

**Published:** 2023-09-14

**Authors:** Katheryn Salow, Helen E. Jack, Joe Tinsley, Caleb J. Banta-Green, Susan Kingston, Matthew Iles-Shih, Judith I. Tsui, Sara Glick

**Affiliations:** 1 School of Medicine, University of Washington, Seattle, USA; 2 Department of Internal Medicine, University of Washington, Seattle, USA; 3 HIV/STI/HCV Program, Public Health–Seattle & King County, Seattle, USA; 4 Department of Psychiatry and Behavioral Sciences, Addictions, Drug and Alcohol Institute, School of Medicine, University of Washington, Seattle, USA; 5 Department of Psychiatry and Behavioral Sciences, University of Washington, Seattle, USA; 6 Division of Allergy and Infectious Diseases, School of Medicine, University of Washington, Seattle, USA

**Keywords:** Syringe service program, Needle exchange, Racial disparities, Demographics, Injection drug use

## Abstract

**Background:**

Syringe service programs (SSPs) provide tools to people who inject drugs (PWID) to prevent overdose, reduce the risk of HIV and HCV infection, and reduce injection frequency. While effective, previous research suggests that SSPs may not adequately reach some marginalized or particularly vulnerable subpopulations of PWID.

**Methods:**

To identify disparities in SSP use, data from two cross-sectional surveys conducted in King County, Washington were compared: a survey of SSP clients and a community survey of PWID in King County. It was hypothesized that Black PWID, women, and gender minorities would be underrepresented in the SSP survey relative to the general population of PWID.

**Results:**

SSP clients identified as White at a significantly higher rate than the community sample of PWID (*p* = 0.030). Black (*p* < 0.001), American Indian/Alaska Native (*p* < 0.001), Latinx/Hispanic (*p* = 0.009), and Native Hawaiian/ Pacific Islander PWID (*p* = 0.034) were underrepresented in the SSP client survey. The gender of SSP clients was similar to the distribution seen in the community sample of PWID (*p* = 0.483).

**Conclusions:**

Black PWID are underrepresented in Seattle-area SSPs, consistent with studies in other large US cities. Both nationally and in Seattle, overdose deaths have been increasing among Black PWID, and harm reduction strategies are vital to reversing this trend. SSPs should explore and test ways to be more accessible to minority populations.

## Introduction

Syringe service programs (SSPs) are an effective harm reduction resource for people who use drugs (PWUD). Services typically include providing sterile injection equipment, offering testing and treatment for communicable diseases, harm reduction education, and connection to treatment and case management for substance use disorder. SSPs have been shown to reduce injection frequency, reduce new HIV and HCV infections, and help individuals with drug cessation [1, 2].

Overdose deaths have been increasing nationally among Black people who inject drugs (PWID), and harm reduction strategies are vital to reversing this trend [3]. Minority PWUD are underserved by many substance use treatment services, including medication for opioid use disorder (MOUD) and harm reduction services [4, 5]. People who identify as Black or Native-Hawaiian/Pacific-Islander/Asian-American have particularly underutilized MOUD [4]. Rosales et al. found that racial/ethnic minorities were ten times more likely to report disrupted access to naloxone and eight times more likely to report difficultly accessing sterile needles during the early COVID-19 pandemic [5]. A qualitative study that interviewed Black community members in the United States found that respondents deemed harm reduction efforts for PWUD inadequate and inappropriate for addressing community need [6]. Women and gender minorities who use drugs also face barriers to accessing harm reduction services. They may experience greater stigma and increased vulnerability relative to their counterparts who identify as men, which can decrease their comfort and perceived safety accessing harm reduction services [7, 8].

Research evaluating who is accessing, or is unable to access, SSPs is scarce. Some studies have looked at the demographics of SSP usage in specific communities in the U.S. Parker et al. investigated the demographics of people who returned to a New York SSP after an initial visit. They found that people who were older, Black, single, or living in unstable housing were less likely to return to an SSP [9]. Similarly, a study conducted at a Miami SSP found that participants were primarily White and not reflective of the demographics of PWID in the predominately African American community of Overtown, where the SSP was located [10]. These studies, however, have only been conducted in specific cities, all of which are on the east coast. Given that drug use and overdose patterns have historically differed across the U.S., with a higher prevalence of methamphetamine use and increased relative rates of opiate overdose deaths on the West Coast, there is a need for additional evaluations to characterize SSP utilization in other regions of the U.S [11, 12].

The aim of this study was to compare the demographics of Public Health – Seattle & King County (PHKSC) SSP clients with a general community sample of PWID in King County, Washington, an urban county that contains Seattle. Based on the existing literature, we hypothesized that individuals who identify as Black will be underrepresented in Seattle-area SSPs relative to the general population of PWID. We also hypothesized that women and gender minorities will similarly be underrepresented. By evaluating potential disparities in SSP use, our results may inform public health efforts and outreach to groups currently underserved by these programs.

## Methods

We utilized two cross-sectional surveys to compare PWID who use a large SSP in King County with the broader population of PWID in King County by using a community-based representative sample of PWID in the region.

### Sample and data collection

#### SSP survey

The 2019 PHSKC SSP survey aimed to include clientele utilizing the PHSKC-run SSP. The survey of SSP participants was conducted at three different program sites: two downtown fixed sites and a mobile van that traveled to South King County. The fixed sites are in very dense areas of downtown Seattle and are easily accessible by public transit. The mobile van is designed to come directly to clients or meet them in an accessible area. At the time of the survey, the SSPs distributed naloxone in addition to sterile injection equipment. The site also offered testing for HIV and viral hepatitis; treatment readiness counseling and case management services; education about harms associated with drug use and how to minimize them; and safe disposal of contaminated equipment. There are two community non-profit run SSPs in Seattle that were not included in these analyses. Over a two-week period in 2019, SSP staff attempted to invite every client seeking services at the SSPs to participate in the survey. SSP survey recruitment took place during SSP Business hours (1–5 pm Monday through Friday). Details of this survey have previously been described in another publication [13]. In summary, it was a venue-based attempted census. The survey took about 10 min to complete, and participants were offered a piece of candy for participation. The inclusion criteria were use of a PHSKC SSP on the day of the survey; there were no exclusion criteria. The survey was anonymous and contained questions about demographics, behavior related to drug use and sex practices, and health conditions.

#### PWID survey

The CDC-funded Seattle-area National HIV Behavioral Surveillance (NHBS) Survey is a behavioral surveillance survey of people at risk for HIV. The aim of the 2018 NHBS-PWID survey was to recruit a representative sample of PWID in the Seattle-area to complete questions on drug use and behaviors, sexual practices, health conditions and demographics. Questions asked in the survey were similar to the SSP survey, though the NHBS-PWID survey was longer (approximately 40 min). The NHBS survey was conducted during traditional business hours (approximately 9am–5 pm Monday through Friday). Similar to the two SSP fixed sites, the NHBS field site was located in a very dense area of Seattle, which was easily accessible by public transit. Further details of survey recruitment were previously published [14]. The survey enrolled participants utilizing respondent-driven sampling, a form of incentivized peer referral which has shown to be an effective method for generating a representative sample of PWUD [15–17]. Eligible participants were people who resided King or Snohomish County, were older than 18, spoke English, and had injected drugs in the past 12 months. Participants received a $50 incentive for completing the survey and were offered a rapid HIV test. They also received an additional $20 for each peer referral.

### Measures

We compared population characteristics between the two surveys. Race and gender were the primary variables of interest. We also evaluated several exploratory variables, including age, self-identification as a man who has sex with men (MSM), housing status, and zip code to help characterize the population of people utilizing SSPs relative to the general population of PWID in King County.

Both surveys utilized a multiple-choice format, and most variables were measured similarly across both surveys. The categories for race included: American Indian/ Alaska Native, Asian/ South Asian, Black/ African American, Latinx/ Hispanic, Native Hawaiian/ Pacific Islander, White and Other. In both surveys, participants could select multiple race groups. The SSP Survey included Latinx/ Hispanic as an option in a question about participant race, while the NHBS-PWID survey asked if patients identified as Latinx/ Hispanic in an ethnicity question separate from selecting racial identification.

The categories for gender included woman, man, transgender, and another gender identity. When asking about gender identity, the SSP survey allowed participants to select one or more than one category from the options: man, woman, genderqueer/ non-binary, trans man, trans woman, and another gender not listed. This differed from the NHBS-PWID survey in that participants could only select one gender of male, female, or transgender. For the analysis, we categorized SSP participants who selected more than one gender identity or genderqueer/ non-binary as “another gender identity” with no comparison group in the NBHS-PWID survey.

Age was calculated based on participants’ reported birthday at the time of survey completion in the NHBS-PWID survey and reported as a number in the SSP survey. In both surveys, MSM was captured using a survey question about the genders of sexual partners in the past 12 months: Individuals who identified as a man in the gender category who indicated they had sex with men in the past 12 months were characterized as MSM. Survey categories for housing status included housed, homeless, or other housing status (e.g., temporary/unstable). For analysis, we collapsed housing status into two groups: currently homeless and other housing status, which included housed individuals. Zip code was a free text numeric entry. For the analysis, we grouped zip codes together into 6 Seattle-area zones: Capitol Hill/ Central District, Downtown, East King County, North Seattle, South King County and South Seattle. The frequency of drug injection was measured in both surveys and recategorized as whether a participant injected daily or not.

### Data analysis

We restricted analyses to participants who reported a valid zip code in King County. We further restricted analyses of the SSP survey data to participants who reported recent injection drug use for comparability with eligibility criteria for the NHBS-PWID survey. NHBS-PWID eligibility included injection drug use in the past 12 months, while the SSP survey only measured injection drug use in the past three months. First, we calculated descriptive statistics of the participant characteristics for each survey group separately. To compare the distribution of the participant characteristics between the SSP survey and the general community sample in NHBS-PWID, we ran chi-squared tests; comparisons that included a cell size of less than five were assessed with Fisher’s exact tests. We conducted two sensitivity analyses. First, we restricted the analysis to participants who injected in the past three months given differences in how this was measured between surveys. Second, given differences in compensation between the two surveys, we restricted the analysis to participants who reported being homeless as a proxy for economic status. All analyses were conducted using Stata 16.0. The 2018 NHBS-PWID survey was determined to be a surveillance project by the Washington State Institutional Review Board (IRB) and did not require review. The University of Washington IRB committee A approved this secondary analysis on 4/21/2021 (IRB ID: STUDY00013026).

## Results

Overall, SSP staff asked 776 people to complete the SSP survey with 432 (56%) agreeing to complete the survey. This is likely an underestimate of the true response rate since people could be approached multiple times, but this could not be tracked in the data. The final analytic sample size for the SSP survey was 368, while the sample size for the NHBS-PWID survey was 531.

### Primary outcomes: race and gender

78% of SSP clients identified as White, which was significantly higher than the proportion of NHBS-PWID survey participants who identified as White (*p* = 0.030). 6% of SSP clients identified as Black, while 19.6% of NHBS-PWID participants identified as Black (*p* < 0.001). Similarly, American Indian/Alaska Native, Latinx, and Native Hawaiian/Pacific Islander PWID comprised a smaller percentage of SSP clients compared to the NHBS-PWID survey participants (*p* < 0.001, *p* = 0.009, and *p* = 0.034, respectively).

Approximately two-thirds of SSP clients were men and one-third were women, with < 1% reporting being transgender and < 1% of clients reporting another gender identity. The gender of SSP clients largely reflected the same distribution seen in a community sample of PWID (*p* = 0.483).

### Secondary outcomes

Among the exploratory demographic characteristics, participants in the SSP survey tended to be younger (mean age: 37.9, SD: 10.4) compared to NHBS-PWID participants (mean age: 41.7, SD: 11.9) (*p* < 0.001). While 48.6% of people in the SSP survey were experiencing homelessness, 60.3% of those of the NHBS-PWID participants were experiencing homelessness (*p* < 0.001). The percentage of MSM participants in the two surveys were similar (*p* = 0.250). A smaller proportion of participants in the SSP survey (67.5%) reported injecting drugs daily compared to the NHBS survey (85.3%, *p* < 0.001).

The percentage of people living in each zip code was different between the two surveys (*p* < 0.001). The largest difference between the two samples was in the proportion of participants in the South King County (i.e., several suburban cities south of Seattle in King County) and South Seattle zip codes. The SSP survey contained more participants from South King County zip codes, while the NHBS-PWID survey contained more participants from South Seattle zip codes.

### Sensitivity analyses

When this analysis was restricted to only NHBS participants who injected in the past three months (*N* = 523/531, 98.5%), the findings were similar to the primary results (data not shown). The only exception was that difference in the proportion of Native Hawaiian / Pacific Islander participants in each survey was only of borderline significance (*p* = 0.056 in the sensitivity analysis). When the analysis was restricted to only participants who were homeless, there was no longer a significant difference in age (*p* = 0.282), Asian race (*p* = 0.960), and Native Hawaiian/Pacific Islander race (*p* = 0.090) between the two surveys. Neither sensitivity analysis resulted in a meaningful change in the findings related to race and gender (Table 1).

**Table 1:** Comparison of SSP users and PWID in King County, WA 2018–2019

Characteristic	SSP Survey Participants 2019	NHBS-PWID Survey Participants 2018	Chi-squared test*p*-value
	*N* = 368 N (%)	*N* = 531 N (%)	*p*-value
Age			< 0.001
18–29	85 (23.1)	93 (17.5)	
30–39	144 (39.1)	157 (29.6)	
40–49	84 (22.8)	123 (23.2)	
50 +	55 (15.0)	158 (29.8)	
Gender^a^			0.483 ^d^
Women	126 (34.2)	201 (37.9)	
Men	238 (64.7)	323 (60.8)	
Transgender	2 (0.5)	7 (1.3)	
Another Gender Identity	2 (0.5)	–	
Race^b^			
American Indian/Alaska Native	43 (11.7)	124 (23.4)	< 0.001
Asian/South Asian	20 (5.4)	14 (2.6)	0.031
Black/African American	22 (6.0)	104 (19.6)	< 0.001
Latinx/Hispanic	27 (7.3)	68 (12.8)	0.009
Native Hawaiian/Pacific Islander	7 (1.9)	24 (4.5)	0.034
White	287 (78.0)	380 (71.6)	0.030
Other	7 (1.9)	–	
Missing	0 (0.0)	9 (1.7)	
Men who have sex with men^c^			0.250
No	198 (83.2)	286 (86.7)	
Yes	40 (16.8)	44 (13.3)	
Housing status			0.001
Currently homeless	179 (48.6)	320 (60.3)	
Other housing status	189 (51.4)	211 (39.7)	
Injects drugs daily			< 0.001
Yes	247 (67.5)	452 (85.3)	
No	119 (32.5)	78 (14.7)	
Zip Code			< 0.001
Capitol Hill/Central District	38 (10.3)	52 (9.8)	
Downtown	129 (35.1)	234 (44.1)	
East King County	30 (8.2)	17 (3.2)	
North Seattle	61 (16.6)	72 (13.6)	
South King County	75 (20.4)	35 (6.6)	
South Seattle	35 (9.5)	121 (22.8)	

^a^SSP participants could select more than one gender, while NHBS participants could only select one gender. SSP participants who selected more than one gender were categorized as Another Gender Identity. Transgender and Another Gender Identity were combined into one category for statistical analysis

^b^Both SSP and NHBS participants could select more than one race/ethnicity

^c^Inclusive of cis and trans men participants

^d^Analyzed using Fisher’s exact test due to cell size < 5

## Discussion

We aimed to evaluate disparities in access to SSPs in an urban county in Washington State. In comparing the demographics between SSP users and a general sample of PWID in the Seattle-area, we found that many PWID from racially minoritized groups were underrepresented in a local SSP. In addition to the hypothesized disparities seen in Black PWID, we found that American Indian/ Alaska Native, Latinx/ Hispanic and Native Hawaiian/ Pacific Islander were also underutilizing SSPs. Notably, Asian/ South Asian and White PWID were overrepresented in the surveyed SSPs. In contrast to the racial/ ethnic findings, the genders of local SSP clients were largely similar to that of a sample of PWID in the region.

This analysis provides further evidence that Black PWID are underrepresented in SSPs. Williams and Metzger found that Black PWUD were significantly more likely to access syringes from non-SSP sites, including works sellers, drug dealers, and other users, which suggested that there may be barriers or deterrents to accessing SSPs among the Black community [18]. Given Black PWUD were more likely to access safe injection equipment from peers, it is possible community-based SSPs could see different trends in client demographics. While our research did not include community-run SSPs, they may be more approachable to marginalized PWUD. Some community-based SSPs set themselves apart by adopting a ‘drug empowerment’ philosophy, which includes hiring peers and engaging with community PWUD to advise on their services. Everson et al. conducted qualitative research among substance use service providers in U.S. communities of color to explore perceptions toward harm reduction services. Researchers concluded that societal racial dynamics played a role in utilization and perceptions of these resources. Interviewees perceived harm reduction as a less-than-ideal approach to substance use disorder. This was thought to be, at least in part, driven by a regime of sociocultural biases and norms derived from the legacy of white supremacy in the US that pressure Black communities to challenge negative stereotypes about race and substance use. In light of this, Black community members may feel that utilizing harm reduction resources could lead to a negative perception of themselves or the Black community as a whole [6].

One other explanation for reduced SSP use among the Black community in the Seattle-area may include the city’s history of segregation. With a history of redlining and racist housing policies that predate the 1960s, Seattle remains a highly racially segregated city with much higher percentages of Black households residing in South King County and South Seattle [19, 20]. A larger distribution of SSP survey participants were from South King County and a smaller distribution were from South Seattle when compared to the community sample of PWID. While PHSKC currently has a mobile SSP van location in South Seattle, the SSPs in this location used to be more robust — with racially diverse staffed sites in Rainier Valley and White Center, however utilization at those sites remained low compared to other sites. Despite funding stresses, PHSKC stayed committed to keeping those sites open specifically to address equity, despite pressure to close low-performing sites and reduce budgets, but over time hours were reduced and closure came even after community relationship-building and advocacy efforts to keep them open. Further investigation is needed to establish how to best address the racial inequities seen in Seattle-area SSPs.

Our findings demonstrate an opportunity to bridge the gap in SSP utilization among minority PWID and additional community-based participatory research may help guide strategies to accomplish this goal. In reviewing previous research we can provide suggestions for ways to begin to address these service disparities. While PHSKC SSPs prioritize recruiting racially diverse staff, there may be room to grow in recruiting more individuals who identify as Black, Latinx, Native American or Pacific Islander. Higher percentages of race/ ethnicity concordance between staff and clients increases the likelihood that clients will access healthcare services [21]. Another way to maximize the accessibly of SSPs is increasing the number of locations and available hours [22]. With increased funding, SSPs could expand operating hours and add additional sites, potentially reaching a more diverse client population [23]. Maintaining anonymity of PWID is also imperative in reducing the stigma associated with accessing an SSP [24]. While the PHSKC SSP did not elicit identifying information from all clients, when clients sought naloxone, they were requested to fill out a form that included their name. This could have inadvertently hindered clients from returning to the SSP given fear of prosecution or judgment.

While other studies have observed that women experience greater stigma related to substance use than men, the fact that women are not underrepresented in the PHSKC SSPs is somewhat surprising and may suggest that this stigma is not a prohibitive barrier to SSP utilization for this group [7]. While we cannot identify why women are not underrepresented among SSP participants, the longevity and reputation of this SSP may explain its ability to maintain an environment that is approachable to woman-identifying PWID. As our surveys sampled very small numbers of SSP clients that identified as gender minorities, we encourage future studies to explore any disparities seen among transgender and nonbinary PWID. For example, interviews with a diverse population of PWID aimed at better understanding women and gender minorities’ perception of SSPs and other harm reduction services could help identify strategies to create an environment that most effectively engages all genders of PWID.

The study had several limitations. There were small differences in question wording and answer choices between the two surveys, which the authors attempted to reconcile to the extent possible. An additional limitation was that both surveys sampled a small number of people who identified as a gender other than man or woman. This makes it difficult to interpret whether disparities might exist for gender identities outside of the binary. Although the NHBS-PWID survey utilized respondent-driven sampling, we did not adjust prevalence estimates using respondent-driven sampling methods in order to facilitate statistical comparisons with the SSP survey data. This analysis also assumes that the NHBS-PWID survey is a representative survey of PWID and uses it as a comparison for assessing which subpopulations are underrepresented in the PHSKC SSP. Due to the fact that the NHBS-PWID survey paid an incentive for both participation and additional recruitment, it may over-enroll people who have lower incomes and are more likely to be homeless. However, in a sensitivity analysis restricted to only participants who were homeless, there was no impact on the findings related to race or gender. There is no truly representative sample of PWID, and the NHBS-PWID survey is the current gold standard. The study focus is limited to use of SSPs to obtain injecting equipment only and does not include information on secondary distribution. Prior research has suggested Black PWID rely more on non-SSP sources for distribution [18].

## Conclusions

Our findings have highlighted that racial disparities in SSP use exist in the Seattle-area. Future research is needed to understand how to best address this disparity. Qualitative research would help explore perceptions of local PWID about SSPs and other harm reduction resources. SSPs are a valuable community resources for PWUD and there is room to grow in building accessible services for all groups.

## Data Availability

The public health surveillance data can be provided upon request and receipt of relevant approvals.

## Abbreviations

SSP: Syringe service program
PWUD: People who use drugs
PWID: People who inject drugs
MOUD: Medication for opioid use disorder
PHSKC: Public Health–Seattle & King County
NHBS-PWID: National HIV Behavioral Surveillance among People Who Use Drugs
MSM: Men who have sex with men
